# Loss of RARRES1 function Promotes Follicular Lymphomagenesis and Inhibits B cell Differentiation in Mice

**DOI:** 10.7150/ijbs.69615

**Published:** 2022-03-28

**Authors:** Jay Patel, Dan Xun, Karen Creswell, Daniel K. Kim, Matthew Wu, Jung-Won Hwang, Taylor S. Kim, Shivani Bansal, Sung-Hyeok Hong, Susana Galli, Hyun-Jung Kim, Chuxia Deng, Stephen W. Byers, Mi-Hye Lee

**Affiliations:** 1 Georgetown-Lombardi Comprehensive Cancer Center, Department of Oncology, Georgetown University Medical Center, Washington, DC, USA.; 2 Flow Cytometry Shared Resource, Georgetown University Medical Center, Washington, DC, USA.; 3 Department of Biochemistry and Molecular & Cellular Biology, Georgetown University Medical Center, Washington, DC, USA.; 4 Department of Molecular Genetics and Dental Pharmacology, School of Dentistry and Dental Research Institute, Seoul National University, Seoul, South Korea.; 5 Faculty of Health Sciences, University of Macau, Macau SAR, China.; 6 Department of Biology and Center for Cell Reprogramming, Georgetown University, Washington, DC, USA.

**Keywords:** RARRES1, B cell, Follicular Lymphoma, UPR, HSR, Metabolism

## Abstract

Retinoic acid receptor responder 1 (RARRES1) is among the most commonly methylated loci in multiple cancers. RARRES1 regulates mitochondrial and fatty acid metabolism, stem cell differentiation, and survival of immortalized cell lines *in vitro*. Here, we created constitutive *Rarres1* knockout (*Rarres1*^-/-^) mouse models to study RARRES1 function *in vivo*. *Rarres1*^-/-^ embryonic fibroblasts regulated tubulin glutamylation, cell metabolism, and survival, recapitulating RARRES1 function in immortalized cell lines. In two mouse strains, loss of *Rarres1* led to a markedly increased dose-dependent incidence of follicular lymphoma (FL). Prior to lymphoma formation, *Rarres1*^-/-^ B cells have compromised activation, maturation, differentiation into antibody-secreting plasma cells, and cell cycle progression. *Rarres1* ablation increased B cell survival and led to activation of the unfolded protein response (UPR) and heat shock response (HSR). *Rarres1* deficiency had differential effects on cellular metabolism, with increased bioenergetic capacity in fibroblasts, and minor effects on bioenergetics and metabolism in B cells. These findings reveal that RARRES1 is a bona fide tumor suppressor *in vivo* and the deletion in mice promotes cell survival, and reduces B cell differentiation with B cell autonomous and non-autonomous functions.

## Introduction

Retinoic acid receptor responder 1 (RARRES1), also known as tazarotene-induced gene 1, was first identified as a retinoid responsive gene in the skin 1. The *Rarres1* promoter region is among the most commonly methylated loci in multiple cancers 2. Prostate cancer and breast cancer cell lines transfected with *Rarres1* are less invasive and more apoptotic, respectively 3,4. Collectively, although rarely mutated in cancers, these data support a role for RARRES1 as a tumor suppressor. The *Rarres1* homolog latexin (*Lxn*) is an established regulator of hematopoiesis. *Lxn-*deficient mice negatively regulate hematopoietic stem and progenitor cell (HSPC) numbers but do not result in B or T-cell tumors 5-7. In zebrafish *Rarres1/Lxn* is also localized to the developing hematopoietic system but its function there is unknown 4.

RARRES1 has dramatic effects on cellular metabolism. In cultured cells RARRES1 can regulate mitochondrial membrane potential, AMPK activation, mTOR and SIRT1 expression, energy balance and metabolically reprograms cells to a more energetic and anabolic phenotype 4,8. *Rarres1* knockdown increases both mitochondrial and glycolytic metabolism. RARRES1 can also control fatty acid metabolism by regulating the switch from aerobic glycolysis to glucose dependent *de novo* lipogenesis, a characteristic of drug resistant B-cell lymphomas 9.

We recently showed that one function of RARRES1 is to inhibit cytoplasmic carboxypeptidase 2 (CCP2), an enzyme that deglutamylates the C-terminal tail of α-tubulin 10. Post-translational modifications of α-tubulin can inhibit flux through voltage-dependent anion-selective channel (VDAC), an outer mitochondrial channel that allows the passage of ATP and other small anions across the outer mitochondrial membrane 11,12. RARRES1 through inhibition of CCP2 can determine α-tubulin post-translational modification, and thus, flux through the VDAC. Functionally, *Rarres1* depletion results in increased metabolic capacity that is associated with the promotion of anoikis, anchorage independent growth and insensitivity to multiple apoptotic stimuli such as chemotherapy.

Here, we generated *Rarres1* knockout (*Rarres1*^-/-^) mouse models to study the effects of RARRES1 loss on cancer development, blood cell compartment function, and cellular metabolism. We discovered that *Rarres1* deletion in two independently derived strains of mice leads to the development of indolent follicular lymphoma (FL) starting around one year of age. With time many mice developed extensive involvement of mesenteric lymph nodes, spleen and liver with a morphology more closely resembling diffuse large B-cell lymphoma (DLBCL), a characteristic of the human disease. DLBCL is the most common type of non-Hodgkin lymphoma and is diagnosed in ~18,000 Americans/year, predominantly in those over 60 years of age. Unlike LXN, RARRES1 loss does not regulate HSPC numbers. However, prior to the development of lymphoma, *Rarres1*^-/-^ B cells had compromised activation, cell cycle progression, and differentiation into antibody-secreting plasma cells. In addition, *Rarres1*^-/-^ B cells showed increased survival, protection from apoptotic cell death, and unfolded protein response (UPR) and heat shock response (HSR) activation. *Rarres1*^-/-^ fibroblasts showed increased bioenergetic capacity, similar to *in vitro* experiments on epithelial cells. These findings reveal that RARRES1 is a bona fide tumor suppressor *in vivo*, the deletion in mice promotes cell survival, and controls B-cell differentiation.

## Materials and Methods

### Mice

*Rarres1*^+/-^ and *Rarres1*^-/-^ mice were generated independently in 129 and C57BL/6N backgrounds. The targeting vector (UC DAVIS KOMP-CSD (ID:39930), PRPGS00072_A_A02) were transfected into 129S1/SvImJ ES cell and the *Rarres1* gene- targeted ES clones were selected and confirmed by long range PCR and Southern blot analysis. Chimeric mice, obtained by injecting the targeted *Rarres1*^+/-^ ES cells into blastocysts, were mated with NIH Black Swiss females (Taconic) to screen for germline transmission. Male mice bearing germline transmission were mated with female B6.FVB EIIa-Cre mice 13 to generate complete global deletion of *Rarres1* exon 3. Detailed ES cell targeting procedures are shown in Figure S1 14 and full-length southern blots are displayed in Supplementary Figures. For C57BL/6N background chimeric mice, KOMP ES cells of Rarres1tm1a(KOMP)Wtsi (UC Davis) were injected by the Transgenic Core Facility of the University of Maryland. All mice were bred and maintained at the Georgetown University Division of Comparative Medicine and experiments were performed according to the NIH guidelines. Mice were genotyped using either southern blot or PCR analysis or the company service from TransnetYX.com, and protocols are available from the authors on request. Mice were monitored for tumor incidence and survival over a period of 2-3 years and were euthanized when visibly ill, according to protocols approved by the Georgetown University Institutional Animal Care and Use Committee.

### Flow cytometry

Cell isolations were handled at 4 °C to minimize cell activation. Bone marrow cells were obtained by crushing tibias and femurs with a mortar and pestle. Cells were gently triturated and filtered through a nylon screen cell strainer (40 μm, Sigma) to obtain a single-cell suspension. Spleen cells were obtained by cutting the spleens into small fragments on a 40 µm cell strainer. Gently, spleen tissue was pressed with pestle (Sigma) and single cells were collected by passing 5 mL of PBS through the cell strainer. Red blood cells were lysed using Red Blood Cell (RBC) Lysis Buffer (Biolegend). Single cell suspensions of spleen or bone marrow were stained with conjugated antibodies for 20 min in 50 µl FACS buffer (2% FBS in PBS) at 4 °C in the dark. Data were obtained on a BD LSRFortessa analyzer (BD Biosciences) and analyzed with FCSExpress 7. Antibodies were used at a 1:200 - 1:500 dilution. Complete antibody information can be found in the Supplemental Methods (Table S1).

### Cell Cycle

For cell cycle analysis, cells were washed twice by centrifugation in PBS at 500 g for 5 min. Then, 10^6^ cells were fixed and permeabilized with 1 mL of ice-cold ethanol overnight at -20 °C. Following two washes with FACS buffer cells were stained in 200 μL of FACS buffer for 30 min at room temperature in the dark with 50 μg/mL Propidium Iodide (PI, Biolegend). Cells were then washed twice with PBS, centrifuged for 5 min at 500 g and re-suspended in 300 μL of PBS. Samples were analyzed on a BD LSRFortessa analyzer (BD Biosciences).

### Apoptosis

For apoptosis analysis, cells were washed twice by centrifugation in PBS at 500 g for 5 min. Cells was resuspended with 100 µl of FACS buffer at a density of 1x10^3^ cells per ml and incubated with 5 µl of FITC-conjugated Annexin V and 5 µl of PI for 15 min at room temperature in the dark. Samples were immediately analyzed by BD LSRFortessa analyzer (BD Biosciences).

### Histology and Immunohistochemistry

Histological and immunohistochemical analyses of lymphoid organs were performed on formalin fixed paraffin embedded (FFPE) tissues, deparaffinized with Xylene, and rehydrated through a graded alcohol series. Heat induced epitope retrieval (HIER) was performed, followed by immunohistochemical staining. Briefly, slides were treated with 3% hydrogen peroxide and 10% normal goat serum for 10 minutes each, and exposed to primary antibodies (Anti-CD45 antibody ab10558 from abcam, CD45R (B220) antibody (RA3-6B2) and CD20 antibody (AISB12) from eBioscience) and subsequently appropriate secondary antibodies and DAB chromogen (Dako). Slides were counterstained with hematoxylin (Fisher, Harris Modified Hematoxylin). Consecutive sections with non-specific IgG of the same isotype as the primary antibody were used as negative controls.

### Activation of isolated mouse B cells

Red blood cell-lysed mouse spleen cells were enriched for B cells using CD43 negative magnetic selection (Invitrogen: 11422D). Cells were grown in RPMI1640 supplemented with 10% FBS, 1% glutamine, 50 μM β-mercaptoethanol, and 1% penicillin-streptomycin. B cells were stimulated with 1 µg/mL anti-Mouse IgM (Jackson ImmunoResearch, 115-006-075), 5 μg/ml anti-CD40 (aCD40) mAb (BioLegend, 102802) and 10 ng/ml IL-4 (BioLegend, 715004).

### RNA extraction and real-time quantitative PCR Analysis

Total RNA was isolated from cells by using Trizol reagent (Invitrogen) or RNeasy kit (Qiagen) according to the instructions of the manufacturer. Reverse transcription was done using Invitrogen Reverse Transcription Kit. The cDNA samples were used to quantify RNA expression using the qRT-PCR performed on a thermocycler (Applied Biosystems) using SYBR green (Roche). Custom primers were made using the Primer-BLAST Primer designing tool from NCBI or from PrimerBank database. Primer sequences can be found in the Supplemental Methods (Table S2). Samples were normalized to MAPK3 or 18S RNA and represented as fold change over control using the ΔΔCT method.

### Immunoblotting

Cells were lysed in RIPA buffer (1% sodium deoxycholate, 0.1% SDS, 1% Triton X-100, 10mM Tris-HCL pH8, 150 mM NaCl supplemented with Protease Inhibitor and Phosphatase Inhibitor Cocktails (Sigma)) for 30 minutes on ice. 10-50 µg protein was separated by 4-12% SDS-PAGE and transferred to nitrocellulose (Amersham Pharmacia Biotech) before blocking in 5% BSA in PBS. Membranes were incubated overnight in the indicated primary antibodies in 1% BSA in PBS: RARRES1 (#HPA003892; Sigma-Aldrich), Δ-2 tubulin (#AB3203; Millipore), Phospho-AMPK (#2535; Cell Signaling), total AMPK (#5831; Cell Signaling), β-Actin (#A5441; Sigma-Aldrich). Blots were washed 3 times for 5 minutes each in PBS- 0.1% Tween 20 (PBST), followed by incubation in HRP-conjugated secondary antibodies (KPL, Gaithersberg, MD) for 1 hour at room temperature on an orbital. Blots were washed 3 times in PBST and the detection of immunoreactive bands was carried out using chemiluminescence with ECL Western Blotting Detection Reagents (Amersham, Piscataway, NJ). Full-length western blots are displayed in Supplementary Figures.

See Supplementary Materials for extracellular flux assay, NAD^+^/NADH detection, targeted metabolomics, and statistical analysis methods.

## Results

### *Rarres1*^-/-^ mice develop high incidence of follicular lymphoma (FL) and exhibit extramedullary hematopoiesis (EMH)

We created global *Rarres1*^-/-^ mice in two different mouse genetic backgrounds: 129S1/SvImJ and C57BL/6N (Figure S1A, S1B). The *Rarres1* gene was disrupted by deleting exon 3 and confirmed by PCR genotyping and Western blot analysis (Figure S1C). Western blot analysis showed increased levels of Δ-2 tubulin in *Rarres1*^-/-^ skin compared to wild type (WT) affirming the *in vitro* function of *RARRES1* as an inhibitor of CCP2 (Figure S1C) 4.

**Figure 1:**
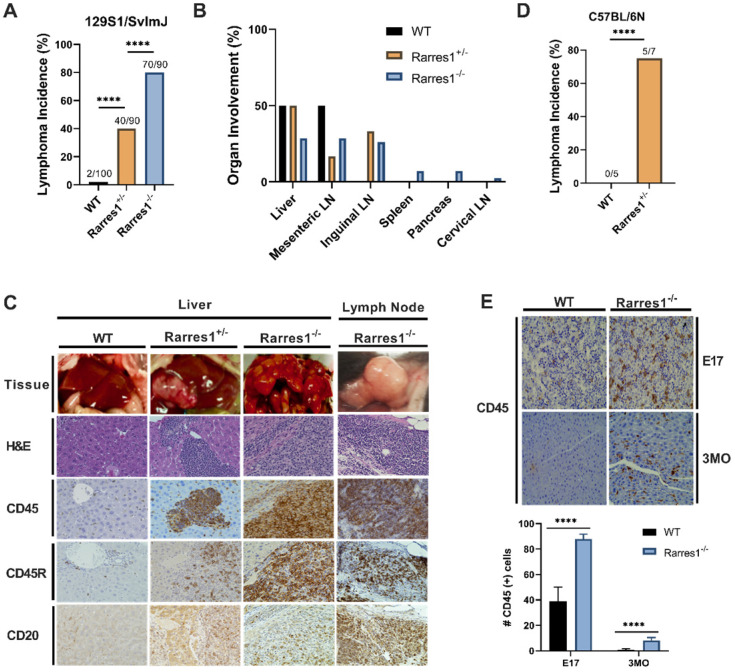
***Rarres1*^+/-^ and *Rarres1*^-/-^ mice develop high incidence of FL. (A)** *Rarres1*^-/-^ mice compared to *Rarres1*^+/-^ and WT mice have a higher life-time incidence of FL development in the 129S1/SvImJ background. Lymphoma arises around 1 year of age in the *Rarres1*^-/-^ mice. P values were calculated by the χ^2^ test. **(B)** FL organ involvement in WT, *Rarres1*^+/-^, and *Rarres1*^-/-^ mice. *Rarres1*^-/-^ mice have widespread organ involvement in the liver, mesenteric LNs, inguinal LNs, spleen, pancreas, and cervical LNs. *Rarres1*^+/-^ and WT do not have as widespread organ involvement pattern as *Rarres1*^-/-^ mice. **(C)** FL in liver and cervical lymph nodes (LNs) of *Rarres1*^-/-^ and *Rarres1*^+/-^ mice compared to normal WT liver. Top panel: whole mount image of liver and cervical LNs (original magnification); middle panel: histological examination by H&E staining (X40); bottom Panel: CD45, CD45R (B220) and CD20 immunostaining (X40). Images acquired and analyzed on Keyence micro-scope (BZ-X). **(D)** *Rarres1*^+/-^ mice in the C57BL/6N background survive and have increased in-cidence of lymphoma as compared to WT mice. *Rarres1*^-/-^ in the C57BL/6N background die perinatally. P values were calculated by the χ^2^ test. **(E)** WT and *Rarres1*^-/-^ mouse liver in the 129S1/SvImJ background were immunostained for CD45 at embryonic day 17 (E17) and 3 months (3MO). *Rarres1*^-/-^ mice contain CD45+ cells in the liver up to 3 MO postnatal, thus exhibiting postnatal extramedullary hematopoiesis.

In the 129S1/SvImJ background, *Rarres1*^-/-^ mice presented at weaning in Mendelian ratio and develop normally with no gross abnormality. However, the life-time incidence of follicular lymphoma was dramatically higher in *Rarres1*^-/-^ mice (70/90, 77.8%) compared to heterozygotes (*Rarres1*^+/-^, 40/90, 44.4%) and WT mice (2/100, 2%) (Figure 1A). Lymphoma onset in the *Rarres1*^-/-^ mice is first detectable around 1 year of age. In some *Rarres1*^-/-^ mice FL displayed widespread organ involvement including the liver, mesenteric lymph nodes (LNs), inguinal LNs, spleen, pancreas, and cervical LNs as indicated by hematoxylin and eosin (H&E) and CD45+, CD45R (B220) +, CD20+ immunohistochemistry (IHC) staining (Figure 1B-C). Because there are multiple isoforms of CD45, some isoforms are specific to T cells and others to B cells, we further checked B cell specific markers such as CD45R (B220) and CD20 (Figure 1C). Pathologist assessment of *Rarres1*^-/-^ FL by H&E staining revealed high grade aggressive FL with characteristics of DLBC lymphoma (Figure 1C). In *Rarres1*^+/-^ mice, FL organ involvement was less widespread and focused in the liver, mesenteric LNs, and inguinal LNs (Figure 1B-C). The dramatically reduced number of lymphomas in wildtype mice involved the liver and mesenteric LNs only (Figure 1B-C). We did not detect increased incidence of epithelial cancers in *Rarres1*^-/-^ mice.

In the C57BL/6N background, *Rarres1*^-/-^ mice die perinatally (2-12h) with no obvious developmental defects, a characteristic of autophagy and metabolism pathway mouse knockouts, consistent with RARRES1 being an important regulator of metabolism. However, *Rarres1*^+/-^ mice in the C57BL/6N background survive, and also develop FL recapitulating what we see in the 129S1/SvImJ background (Figure 1D).

Additionally, in the 129S1/SvImJ background, *Rarres1*^-/-^ mice show survival of CD45+ cells within the liver up to 3 months postnatally suggesting extramedullary hematopoiesis (EMH) even after birth (Figure 1E) 15. The FL and EMH phenotypes indicate that RARRES1, like its homolog *Lxn*, is important for normal homeostasis of the blood cell compartment. Since our *Rarres1*^-/-^ mice were constitutive in both backgrounds, we could not determine if the FL lymphomagenesis phenotypes were driven by B cell intrinsic or B cell extrinsic effects. To address this, we next investigated the role of RARRES1 on B-cells isolated from WT and *Rarres1*^-/-^ mice.

### Mature blood cell and HSPC numbers are normal in *Rarres1*^-/-^ mice

**Figure 2:**
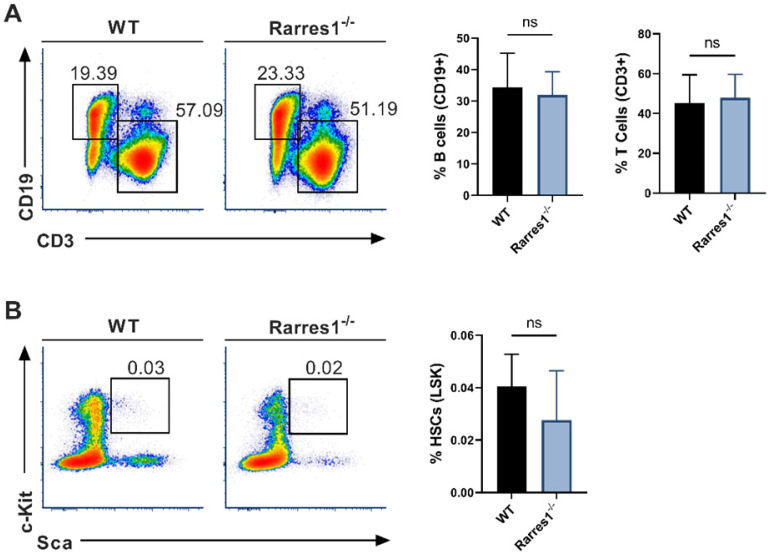
** Mature lymphocyte cell and HSC numbers are normal in *Rarres1*^-/-^ mice. (A)** Flow cytometry percentage of B cells (CD19+) and T cells (CD3+) from WT (n = 5) and *Rarres1*^-/-^ (n = 5) spleen. **(B)** Flow cytometry percentage of HSCs (Lin-Sca1+cKit+) from WT (n = 5) and *Rarres1*^-/-^ (n = 5) bone marrow. Data shown as mean (±SD). Significance was determined by unpaired two-tailed t-test; *P < 0.05; **P < 0.01; ***P < 0.001; ****P < 0.0001.

As knockout of *Lxn* in mice increases the numbers of HSPCs and mature blood cells, we wanted to know if *RARRES1* loss could increase the numbers of B cells before FL development thereby increasing the risk for lymphoma development. Using flow cytometry, we analyzed the level of mature blood cells in WT and *Rarres1*^-/-^ mouse spleen of young mice aged 0-6 months. There was no change in T cells (CD3+) and B cells (CD19+) (Figure 2A). The numbers of macrophages (CD11b/Mac-1+), granulocytes (Mac-1/Gr-1+), NK cells (CD335+), and plasma cells (CD138+) were also unchanged (supplemental Figure 2A). To see if *Rarres1*^-/-^ mice, like *Lxn* knockout mice, had an effect on the levels of blood stem cells, we assessed the levels of HSCs (Lin-Sca1+cKit+), multipotent progenitor cells (MMP; Lin-Sca1+cKit+CD34-CD135/FLT3/Flk2+), and common lymphoid progenitors (CLP; Lin- I17rα+cKit+Sca1+) in the bone marrow using flow cytometry, and observed no difference between *Rarres1*^-/-^ and WT mice (Figure 2B and Figure S2B).

### GC and plasma cell differentiation and antibody production are decreased in *Rarres1*^-/-^ B cells

**Figure 3:**
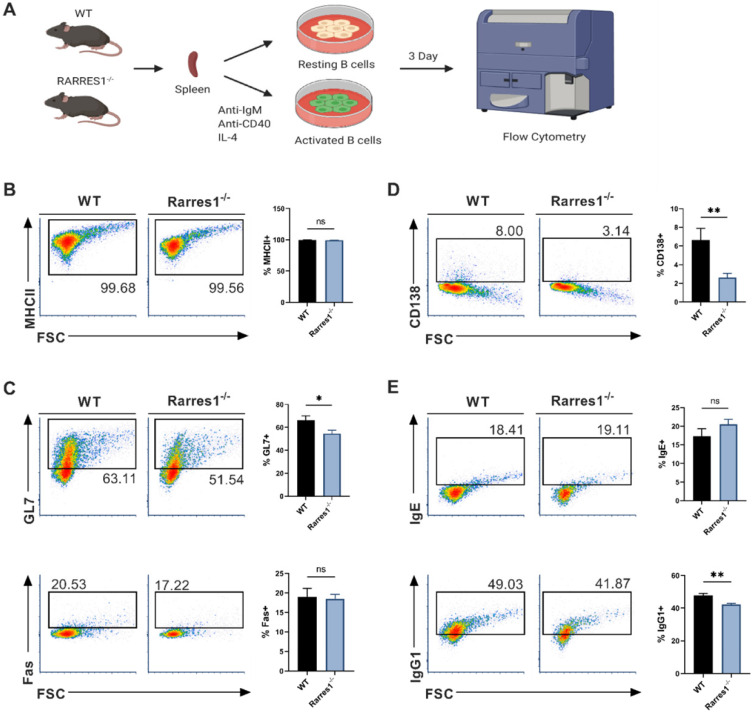
** GC and plasma cell differentiation and antibody production are decreased in *Rarres1*^-/-^ B cells. (A)** Illustration of TD *in vitro* B cell activation study design. **(B)** Flow cytometry analysis for activation marker MHCII from WT (n = 3) and *Rarres1*^-/-^ (n = 3) B cells after 3 days of TD activation. **(C)** Flow cytometry analysis for GC differentiation markers Fas and GL7 from WT (n = 3) and *Rarres1*^-/-^ (n = 3) B cells after 3 days of TD activation. **(D)** Flow cytometry analysis for plasma cell marker CD138+ from WT (n = 3) and *Rarres1*^-/-^ (n = 3) B cells after 3 days of TD activation. **(E)** Flow cytometry analysis for CSR markers IgG1 and IgE from WT (n = 3) and *Rarres1*^-/-^ (n = 3) B cells after 3 days of TD activation. Data shown as mean (±SD). Significance was determined by unpaired two-tailed t-test; *P < 0.05; **P < 0.01; ***P < 0.001; ****P < 0.0001.

Since there was no change in the level of B cell numbers in the blood and spleen of *Rarres1*^-/-^ mice prior to lymphoma formation, it is not simply the higher production of B cells that accounts for FL development. We next explored how *RARRES1* loss leads to change in B cell intrinsic function before the onset of FL. FL is a cancer of activated B cells residing in the peripheral lymphoid organs that incompletely differentiate, thus we sought to understand how *Rarres1* deletion affects B cell activation and differentiation as it does in other cell types* in vitro* 16. To do so, we modeled B cell activation *in vitro* allowing us to study how B cell intrinsic loss of *RARRES1* leads to dysfunction. Splenic B cells from WT and *Rarres1*^-/-^ mice were isolated and activated *in vitro* by stimulation with anti-IgM (aIgM), anti-CD40 (aCD40), and IL-4, thus modeling T cell-dependent (TD) activation of B cells (Figure 3A) 17. After stimulation, B cells enter the cell cycle, proliferate, and differentiate into germinal center (GC) B cells and plasma cells. Differentiation into GC B cells facilitates immunoglobulin gene somatic hypermutation (SHM) and class-switch recombination (CSR), two essential processes for making high affinity and class diverse antibodies. Differentiation into plasma cells is necessary for B cells to become professional antibody-secreting cells. After 3 Days of TD activation, *Rarres1*^-/-^ B cells had similar expression of MHCII, a marker for activated B cells, and GC marker Fas, but expression of GC marker GL7 declined (Figure 3B-C). In addition, plasma cell differentiation, reflected by CD138 expression, and antibody IgG1 production were also reduced in *Rarres1*^-/-^ B cells after 3 days of TD activation, while no change was observed in antibody IgE production (Figure 3D-E).

The defect in plasma cell differentiation was unexpected as we did not observe a change in plasma cell numbers in *Rarres1*^-/-^ spleen (Figure S2A). We hypothesize two reasons for this outcome. First, the mice are housed in sterile environments, and thus with minimal exposure to pathogens, the presence of short-lived plasma cells in the spleen would be uncommon. Second, the *in vitro* assay likely has a higher sensitivity for determining the defects of differentiation capacity as large numbers of B cells are forced to differentiate; therefore, differences between *Rarres1*^-/-^ and WT differentiation are more easily measured.

Overall, RARRES1 loss decreased GC B cell and plasma cell formation, and decreased the functional ability of B cells to produce antibodies. Basically, *Rarres1*^-/-^ B cells are maintained in a less differentiated state.

### *Rarres1* ablation impedes cell cycle progression and enhances B cell survival

**Figure 4:**
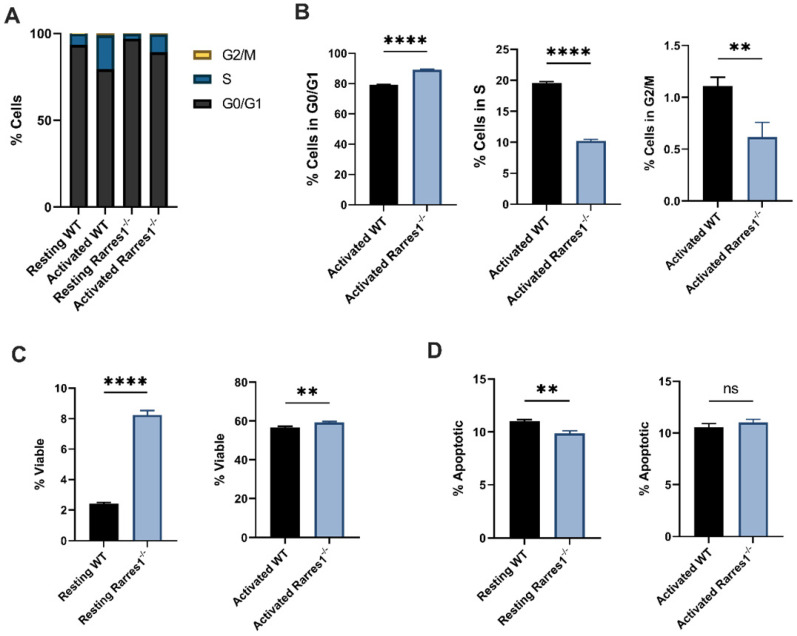
***Rarres1* ablation impedes cell cycle progression and enhances B cell survival. (A)** Flow cytometry analysis of cell cycle phases (G0/G1, S, G2M) from WT (n = 3) and *Rarres1*^-/-^ (n = 3) B cells after 3 days of TD activation. **(B)** Quantification of cell cycle phases differences between WT (n = 3) and *Rarres1*^-/-^ (n = 3) B cells after 3 days of TD activation. **(C)** Flow cytometry analysis of viability (PI staining) from WT (n = 3) and *Rarres1*^-/-^ (n = 3) resting and activated B cells after 3 days. **(D)** Flow cytometry analysis of apoptosis (Annexin V staining) from WT (n = 3) and *Rarres1*^-/-^ (n = 3) resting and activated B cells after 3 days. Data shown as mean (±SD). Significance was determined by unpaired two-tailed t-test; *P < 0.05; **P < 0.01; ***P < 0.001; ****P < 0.0001.

We next determined if RARRES1 influences cell cycle progression. Compared to WT, *Rarres1*^-/-^ B cells accumulated at the G0/G1 phase of the cell cycle (Figure 4A) after 3 days of TD activation. Thus, there was an increased number of *Rarres1*^-/-^ B cells in G0/G1 and fewer in S and G2/M compared to WT (Figure 4B). The decrease in cell cycle progression is likely the result of impaired differentiation into GC B cells and plasma cells.

*Rarres1* transient and stable knockdown *in vitro* in immortalized cell lines protects from cell death induced by multiple damaging agents 4. Similarly resting (unstimulated) *Rarres1*^-/-^ B cells were significantly more viable, determined by propidium iodide staining, than WT after 3 days in culture (Figure 4C). The 3-day TD activated *Rarres1*^-/-^ B cells show only a marginal increase in viability (Figure 4C). This result may be caused by the fact that artificial *in vitro* activation can lead to maximal stimulation of the B cells that masks subtle, but phenotypically important, RARRES1-dependent effects on viability. The resting *Rarres1*^-/-^ B cells underwent slightly decreased levels of apoptosis compared to WT (Figure 4D), but the magnitude of protection from apoptosis was not commensurate with the increase in cell viability. Thus, inhibition of apoptotic cell death is not the only mechanism by which the *Rarres1*^-/-^ B cells retain viability in culture. However, we showed previously that RARRES1 also influences other modes of cell death including autophagy 8. Consistent with a role for RARRES1 on cell death, the effects of *Rarres1* ablation on lymphoma development, B-cell differentiation and survival are very similar to those observed earlier in the *Bad* KO mouse 18.

### *Rarres1* inactivation activates UPR and HSR

Given that decreased apoptosis cannot fully account for the increased viability of resting *Rarres1*^-/-^ B cells, we next looked at other potential explanations. We assessed the roles of the Unfolded Protein Response (UPR) and the Heat Shock Response (HSR), two conserved systems that help cells adapt to disruption in cellular homeostasis and influence autophagy 19. In fact, X-box binding protein 1 (XBP1), a transcription factor that drives activation of the UPR, is necessary for B cell differentiation into long-lived plasma cells and sustained antibody production 20,21.

The endoplasmic reticulum (ER) is needed for the proper folding and maturation of newly synthesized secretory and transmembrane proteins. Accumulation of unfolded proteins in the ER results in cell stress that leads to activation of the UPR 22. Activation of the UPR can be measured by increased gene expression of UPR effector genes, such as, activating transcription factor 4 (ATF4), C/EBP homologous protein (CHOP), binding immunoglobulin protein (BiP), ER degradation enhancing mannose-like protein 1 (EDEM1), and spliced XBP1 (sXBP1) 23.

Various stressors such as heat, reactive oxygen species, and heavy metals can cause protein damage and the aggregation of unfolded proteins. Upon such protein stress, the HSR activates and the expression of chaperone proteins that help refold proteins increases 24. Activation of the HSR can be measured by increased expression of HSR chaperones including HSP90A, HSP90B, HSP70-1A, HSP70-1B, HSPA14.

**Figure 5:**
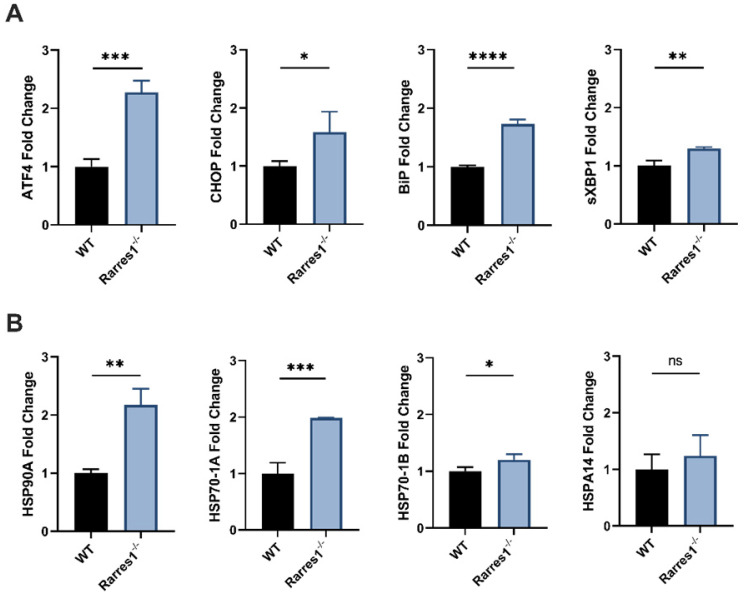
***Rarres1* inactivation increases basal levels of UPR and HSR. (A)** qPCR expression of UPR genes ATF4, CHOP, BiP, and sXBP1 of resting WT (n = 3) and *Rarres1*^-/-^ (n = 3) B cells after 3 days of culture. **(B)** qPCR expression of HSR genes HSP90A, HSP70-1A, HSP70-1B, and HSPA14 in 3 days resting WT (n = 3) and *Rarres1*^-/-^ (n = 3) B cells after 3 days of culture. Data shown as mean (±SD). Significance was determined by unpaired two-tailed t-test; *P < 0.05; **P < 0.01; ***P < 0.001; ****P < 0.0001.

To determine if the increase in cell viability of resting *Rarres1*^-/-^ B cells is in part due to increased UPR and HSR activation, we assessed if resting *Rarres1*^-/-^ B cells increase expression of UPR and HSR activation genes after 3 days in culture. *Rarres1*^-/-^ B cells have a two-fold increase in expression of the UPR regulators ATF4, CHOP, and BiP (Figure 5A). sXBP1 expression increases slightly (Figure 5A). For the HSR, there is increased expression of HSP90A, HSP70-1A, and HSP70-1B, but levels of HSPA14 do not change (Figure 5B). *Rarres1*^-/-^ B cells, therefore, activate UPR and HSR, which can increase resistance to protein and cell stress, thereby increasing the threshold for cell death resulting in higher cell viability even under conditions of stress.

### RARRES1 does not significantly influence B cell OCR and ECAR but does increase bioenergetic capacity of MEFs

A growing body of evidence suggests that some types of lymphoma are more reliant on fatty acid driven oxidative phosphorylation (OXPHOS) for ATP production 25. The exact role of OXPHOS on lymphoma development is unclear, but targeting OXPHOS pathways produce therapeutic benefit in preclinical models 26,27.

**Figure 6:**
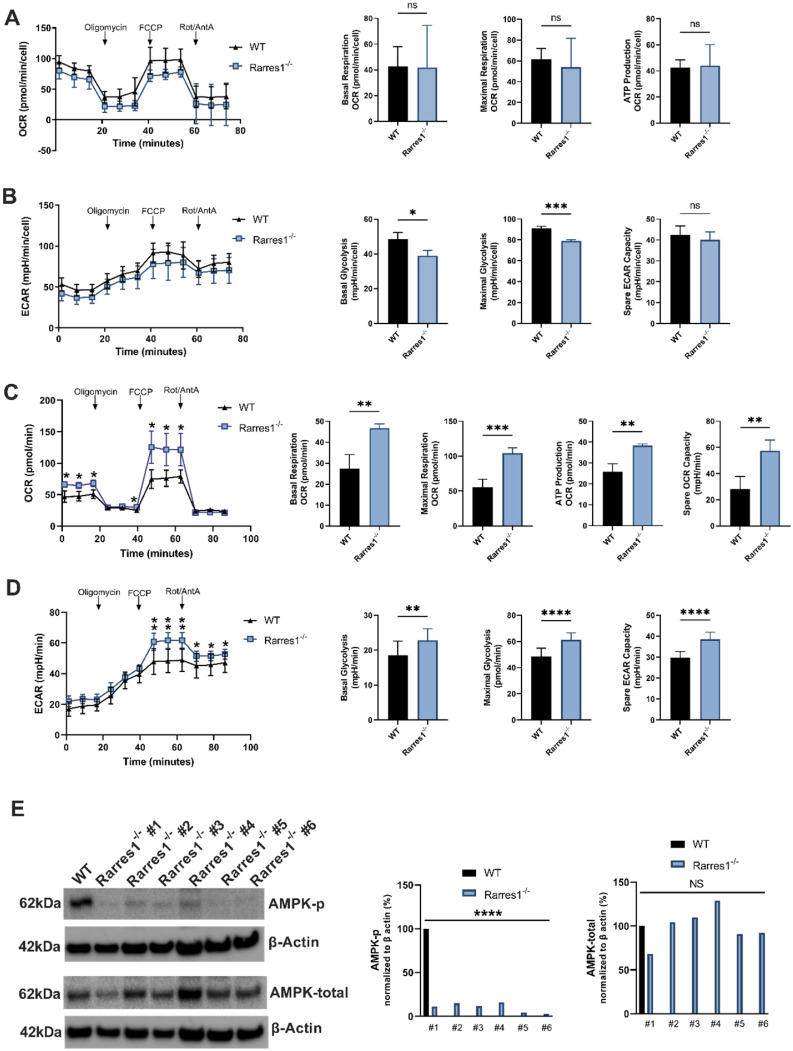
***Rarres1* does not significantly influence B cell OCR and ECAR but does increase bioenergetic capacity of MEFs. (A)** OCR over time with sequential addition of oligomycin (1 µM), mitochondrial uncoupler FCCP (2 µM), and electron transport inhibitors antimycin (1 µM) + rotenone (1 µM), in WT (n = 3) and *Rarres1*^-/-^ (n = 3) B cells after 24 hr TD activation. Differences in basal OCR, maximal respiration, and ATP production were quantified. **(B)** ECAR over time with sequential addition of oligomycin (1 µM), mitochondrial uncoupler FCCP (2 µM), and electron transport inhibitors antimycin (1 µM) + rotenone (1 µM), for WT (n = 3) and *Rarres1*^-/-^ (n = 3) B cells after 24 hr TD activation. Differences in basal glycolysis, maximal glycolysis, and glycolytic capacity were quantified. **(C, D)** WT and *Rarres1*^-/-^ MEF OCR and ECAR were determined by extracellular flux analysis (n = 4). OCR/ECAR over time with sequential addition of oligomycin (1 µM), mitochondrial uncoupler FCCP (1 µM), and electron transport inhibitors antimycin (1 µM) + rotenone (1 µM). **(E)** Western blot analysis of P-AMPK and total AMPK from WT and *Rarres1*^-/-^ MEF cells. The densitometry intensity of each band was normalized to β-actin level. Data shown as mean (±SD). Significance was determined by unpaired two-tailed t-test; *P < 0.05; **P < 0.01; ***P < 0.001; ****P < 0.0001.

Given that RARRES1 can have a profound effect on cellular metabolism and OXPHOS in other systems, we explored RARRES1 effects on metabolism in B cells 9. However, *Rarres1*^-/-^ B cells do not alter mitochondrial OXPHOS after 24 hrs of TD activation. In B cells, RARRES1 loss tends to decrease metabolism, though not statistically significantly for basal OCR, maximal respiration, and ATP production (Figure 6A). Basal glycolysis and maximal glycolysis, measured by extracellular acidification rate (ECAR), decrease modestly in *Rarres1*^-/-^ B cells (Figure 6B). Spare glycolytic capacity, which is reflected as maximal glycolysis subtracted by basal glycolysis, tends to decrease, but the changes are modest as well (Figure 6B). One caveat in these experiments is that the isolated B-cells, unlike fibroblast and epithelial cell lines, do not survive well in the conditions required for the Seahorse assay.

To complement the seahorse experiments, we performed targeted metabolomics experiments in 24 hr TD activated WT and *Rarres1*^-/-^ B cells. Flux toward glycolysis and TCA cycle declined in *Rarres1*^-/-^ B cells, but the changes were modest and not statistically significant (supplementary Figure 3A-B). Glutamine and glutamate levels tended toward increasing in *Rarres1*^-/-^ B cells, suggesting increased glutaminolysis (Figure S4A) 28. Reduced glutathione (GSH) increased, while oxidized glutathione (GSSG) decreased, but not significantly, in *Rarres1*^-/-^ B cells (Figure S4B) 29,30. The potential for an increased GSH/GSSG ratio suggests a greater capacity to scavenge ROS, and possibly, the increased viability of resting *Rarres1*^-/-^ B cells could be due to increased GSH antioxidant capacity. Last, *Rarres1*^-/-^ B cells show a small but significant decrease in the levels of nicotinamide adenine dinucleotide (NAD+), which could have implications in regard to signaling and DNA repair as Poly (ADP-ribose) polymerases (PARPs) and SIRTuins (SIRTs) consume NAD+ in order to function (Figure S4C) 31,32.

On the other hand, *Rarres1*^-/-^ mouse embryonic fibroblasts (MEFs) have significantly increased levels of basal/maximum oxygen consumption (OCR), ATP production and spare respiratory capacity (SRC), which recapitulates the effects of *Rarres1* knockdown in immortalized cell lines (Figure 6C) 4,9. Basal/maximal glycolysis and Spare glycolytic capacity, measured by extracellular acidification rate (ECAR), have also increased significantly in *Rarres1*^-/-^ B cells (Figure 6D). Furthermore, *Rarres1*^-/-^ MEFs have decreased levels of phosphorylated-AMP-activated protein kinase (P-AMPK) as it does in immortalized cell lines (Figure 6E). Metabolic energy deprivation can be sensed by AMPK via changes in the ATP-to-AMP ratio within cells. High AMP relative to ATP causes phosphorylation, and thus activation, of AMPK 33. Activated AMPK reprograms metabolism so that pathways that produce ATP are activated (e.g. mitochondrial biogenesis and autophagy) and pathways that consume ATP (e.g. lipid synthesis and gluconeogenesis) are switched off. The increase in energy capacity of *Rarres1*^-/-^ MEFs to make ATP results in diminished activation of AMPK as cellular ATP levels are more than plentiful. However, since we did not see any major changes in oxidative or glycolytic metabolism in *Rarres1*^-/-^ B-cells, it is highly unlikely that AMPK phosphorylation was altered in these cells.

Taken together, in contrast to *Rarres1*^-/-^ fibroblasts and immortalized epithelial cells, these data indicate that B cells isolated from *Rarres1*^-/-^ mice do not substantially alter their global metabolism and energetics compared to WT B cells.

## Discussion

Previous studies indicated that RARRES1 has tumor suppressor characteristics. Methylation sequencing revealed that the *Rarres1* promoter was commonly methylated in many different cancers and expression of RARRES1 was decreased in a variety of cancers 2,34. Cell culture studies in immortalized cell lines showed that Rarres1 depletion augments many cancer phenotypes including: protection from apoptosis and anoikis, anchorage independent growth, and autophagy induction 3,4,8. Furthermore, RARRES1 has significant effects on stem cell populations and phenotypes and its loss is often associated with dedifferentiation 4. However, *in vivo* evidence of RARRES1 acting as tumor suppressor was lacking.

In this study, we show that constitutive *Rarres1* ablation leads to a high incidence of indolent FL, with a disease course that is remarkably similar to the human disease, including the presence of DLBC-like lymphomas at multiple sites in many animals. This dramatic phenotype validates RARRES1 as a tumor suppressor *in vivo* and is consistent in two independently derived strains of mice. We did not detect increased penetrance of epithelial cancers such as breast, prostate, or pancreatic cancer although these solid tumors usually acquire mutations in oncogenes to become transformed. Future experiments will explore the role of* Rarres1* loss in the context of organ specific oncogenic mutations. In contrast, in both aged mice and humans up to 70% of normal B-cells harbor the bcl2 or myc translocations common in FL, strongly indicating that other modifiers are necessary to convert these cells into FL. It is possible that altered expression or function of RARRES1 or the metabolic pathways it regulates play a role in the age dependent onset of FL as well as its resistance to therapy. Since we used whole-body *Rarres1* knockout mice, our study does not rule out an additional role of RARRES1 loss in affecting the microenvironment of the hematopoietic niche, and thus, influencing FL development. Future studies that generate B cell and niche specific *Rarres1* knockout mice will be essential to decipher B cell intrinsic from the extrinsic functions of RARRES1. Although we did investigate the function of WT and *Rarres1*^-/-^ B cell preparations isolated prior to lymphoma development, these were short term experiments and do not formally rule out a role for a prior influence of the *Rarres1^-/-^* niche cells.

Consistent with the known origins of FL, TD-activated B cells depleted of Rarres1 are less able to undergo maturation and differentiation, indicated by decreased expression of GL7 and CD138. As FL arises from immature and undifferentiated GC B cells, *Rarres1* loss can promote FL by obstructing B cell maturation and differentiation 35,36. Furthermore, cell cycle progression *in vitro* is reduced in *Rarres1-*KO B cells, which is likely the result of decreased maturation and differentiation.

The viability of resting *Rarres1*^-/-^ B cells increases while in culture. *Rarres1*^-/-^ cells increase activation of the UPR and HSR, which may explain the increased hardiness and viability of the cells. The increased hardiness of the resting B cells may also account for increased FL development due to increased B cell survival of undifferentiated B cells in the periphery. A recent study discovered that chaperone mediated autophagy (CMA), the process of chaperone-guided selective degradation of proteins by the lysosome, is important for HSC survival, self-renewal, and activation 37,38. Loss of CMA resulted in compromised protein quality control and prevented the upregulation of fatty acid metabolism upon HSC activation. Possibly, the increase in UPR and HSR can activate CMA in undifferentiated resting *Rarres1*^-/-^ B cells in the periphery thereby serving as a mechanism for increased B cell survival. As CMA can influence fatty acid metabolism, it would be interesting to determine if resting *Rarres1*^-/-^ B cells or *Rarres1*^-/-^ niche cells deregulate fatty acid metabolism, which would align with RARRES1's role as a mediator of cellular metabolism, and specifically, of fatty acid metabolism 4,9.

*Rarres1*^-/-^ MEFs show an increase in cellular bioenergetic capacity similar to that observed in immortalized cell lines following transient or stable depletion of *Rarres1* 4,9. As the *Rarres1*^-/-^ MEFs do exhibit increased metabolism, it is possible that the metabolism of niche cells is influenced by *Rarres1* ablation. Indeed, there is a growing realization in other systems that alterations in the metabolism of cancer-associated stromal cells and immune cells can profoundly influence the growth of cancer cells 39,40. The future development of niche specific knockouts of *Rarres1* will help answer such questions.

**Figure 7:**
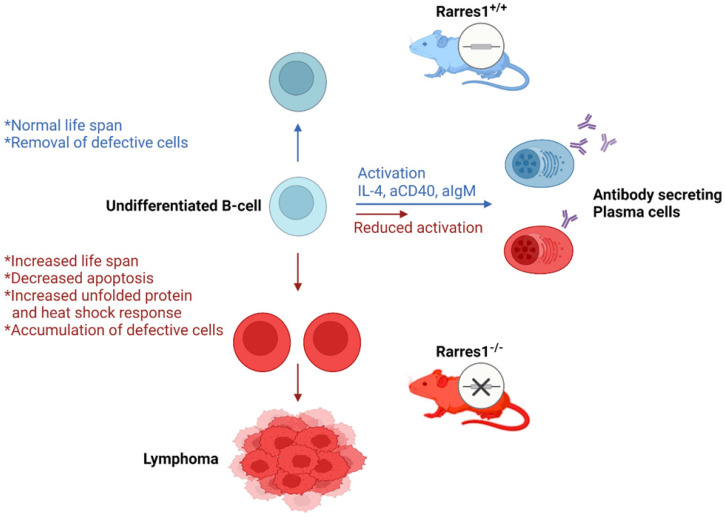
** Proposed model of follicular lymphomagenesis and B cell differentiation in *Rarres1* Knockout mice.** (left side of figure) Somatic deletion of *Rarres1* results in high penetrance of diffuse large B-cell lymphoma in two independently derived strains of mice. (right side of figure) Prior to lymphoma development, isolated *Rarres1*^-/-^ B-cells exhibit reduced activation to plasma cells and increased survival. Undifferentiated *Rarres1*^-/-^ B-cells have lower levels of apoptosis and increased unfolded protein and heat shock responses.

Previous research on RARRES1 indicated that it acted as a tumor suppressor in multiple cancers. *Rarres1* depletion in immortalized cell lines promoted anchorage-independent growth and insensitivity to multiple apoptotic stimuli. While RARRES1 is epigenetically silenced in many cancers, *in vivo* validation of RARRES1 as a tumor suppressor was lacking. Here, we have discovered that genetic knockout of Rarres1 in mice leads to almost complete penetrance of follicular lymphoma. As summarized in Figure 7, mechanistically, we show that *Rarres1* knockout compromises B cell activation and differentiation into antibody-secreting plasma cells. Loss of *Rarres1* in B cells leads to enhanced survival, associated with decreased apoptosis and activation of the unfolded protein and the heat shock responses. Significantly, *Rarres1* knockout affects CCP2 dependent tubulin glutamylation and increased bio-energetic capacity in mouse embryonic fibroblasts, but not in B cells, suggesting that RARRES1 is acting within the tumor microenvironmental niche as well. This work advances the knowledge of the molecular underpinnings of follicular lymphoma, which is a complex process involving somatic gene mutations, chromosomal translocations, and epigenetic alterations. Our work suggests that the well-known age-dependent epigenetic silencing of RARRES1 expression can enhance follicular lymphoma development.

## Supplementary Material

Supplementary methods, figures and tables.

## Abbreviations

RARRES1: Retinoic acid receptor responder 1
FL: follicular lymphoma
EMH: extramedullary hematopoiesis
LXN: Latexin
HSPC: hematopoietic stem and progenitor cell
CCP2: cytoplasmic carboxypeptidase 2
VDAC: voltage-dependent anion-selective channel
MEFs: mouse embryonic fibroblasts
FFPE: formalin fixed paraffin embedded
HIER: heat induced epitope retrieval
H&E: hematoxylin and eosin
IHC: immunohistochemistry
LNs: lymph nodes
aIgM: anti-IgM
aCD40: anti-CD40
GC: germinal center
SHM: somatic hypermutation
CSR: class-switch recombination
UPR: unfolded protein response
HSR: heat shock response
ER: endoplasmic reticulum
XBP1: X-box binding protein 1
sXBP1: spliced XBP1
ATF4: activating transcription factor 4
CHOP: C/EBP homologous protein
BiP: Binding immunoglobulin protein
EDEM1: ER degradation enhancing mannose-like protein 1
OXPHOS: oxidative phosphorylation
SRC: spare respiratory capacity
ECAR: extracellular acidification rate
AMPK: adenosine monophosphate-activated protein kinase
NAD: nicotinamide adenine dinucleotide
PARPs: Poly (ADP-ribose) polymerases
SIRTs: SIRTuins
CMA: chaperone mediated autophagy
