# Supplementary material for: Loss of RARRES1 function Promotes Follicular Lymphomagenesis and Inhibits B cell Differentiation in Mice
Source: Int J Biol Sci. 2022 Mar 28;18(7):2670–82. doi: 10.7150/ijbs.69615 (PMC9066096; doi:10.7150/ijbs.69615)

Loss of RARRES1 function Promotes Follicular Lymphomagenesis and Inhibits B cell Differentiation in Mice

Jay Patel[1], Dan Xu[2], Karen Creswell[2], Daniel K. Kim[1], Matthew Wu[1], Jung-Won Hwang[1], Taylor S. Kim[1], Shivani Bansal[1], Sung-Hyeok Hong[3], Susana Galli[3], Hyun-Jung Kim[4], Chuxia Deng[5], Stephen W. Byers[1✉], and Mi-Hye Lee[1,6✉]

Supplementary Methods

Metabolic analysis using extracellular flux assays
Bioenergetics profile of WT and *Rarres1*$^{-/-}$ cells were measured using the XF^{e}96 Extracellular Flux Analyzer. The day of the assay, the cells were seeded at an optimized cell density (400,000 cells/well) in the 96-well XF^{e} plate and incubated at 37˚C in a CO_2-free atmosphere incubator for 1 hour. Oxygen consumption rates (OCRs) and extracellular acidification rates (ECARs) and response to mitochondrial stress factors were analyzed by using the XF Cell Mito Stress Kit. Basal OCR and ECAR were measured. Subsequently, OCR and ECAR responses were observed after sequential injections of oligomycin (1 µM), carbonyl cyanide-ptrifluoromethoxyphenylhydrazone FCCP (2 µM), and a combination of rotenone and antimycin A (1 µM). For each injection, there were a total of 3 cycles, each one lasted 3 minutes and a measurement was taken at the beginning of each cycle.

NAD^+/NADH Metabolite Detection
NAD^+ and NADH levels were quantified by Promega NAD/NADH-Glo bioluminescence assay according to the manufacturer's instructions (Promega G9071).

LC-MS metabolite extraction, targeted LC-MS conditions, and polar metabolites analysis
The cell samples thawed in ice at room temperature. Cells were re-suspend in 50 µL of chilled Water/Methanol/IPA (35:25:40). Samples were plunged into dry ice for 30 seconds and heat shocked in a water bath for 90 sec. Repeat freeze thaw cycles were performed and then samples were sonicated 30 sec. Chilled methanol containing internal standards (500 ng/mL of Debrisoquine and 500 ng/mL of Taurine d-4) were added to samples. Samples were vortexed and kept it on ice for 20 min, incubated at -20 °C for 20 min, and then centrifuged at 13000 rpm for 20 min at 4 °C. Supernatant was transferred to MS sample vial capped, and run on LC-MS machine.

Each sample (5 µL) was injected onto a Kinetex 2.6 µm 100 Å 100 × 2.1 mm (Phenomenex) column with a triple quadrupole mass spectrometer (5500 QTRAP, SCIEX, USA) operating in the multiple reaction monitoring (MRM) mode. The gradient mobile phase comprised of: solvent A-100% water + 0.2% Formic acid; solvent B-100% ACN + 0.2% Formic acid. Each sample was resolved for 22.5 min at a flow rate of 0.2 mL/min. The gradient consisted of 100% A and 0% B for 2.10 minutes, then 5% A and 95% B from 14 min. to 15.10 min., 2% B and 98% D to 9.0 min., a hold of 2% B and 98% D up to 15.10 min, and then 100% A and 0% B from 15.10 min to 22 min 11.5 min. The column eluent was introduced directly into the mass spectrometer by electrospray.

The declustering potential, collision energies, cell exist potential, entrance potential were optimized for each metabolite to obtain maximum ion intensity for parent and daughter ions via manual tuning in Analyst 1.6.3 software (SCIEX, USA). Signal intensities from all MRM Q1/Q3

ion pairs for the analyte were ranked to ensure selection of the most intense precursor and fragment ion pair for MRM-based quantitation. This approach resulted in selection of declustering potential, collision energies, cell exist potential, entrance potential that maximized the generation of each fragment ion species. The metabolite ratios were calculated by normalizing the peak area of endogenous metabolites within samples normalized to the internal standard debrisoquine (DBQ) for positive mode metabolites and taurine-d4 for negative mode metabolites. The sample queue was randomized and solvent blanks were injected to assess sample carryover. Pooled QC samples were injected after every six samples to check for instrumental variation. Data was normalized to QC variance. MRM data were processed using MultiQuant 3.0.3. The relative quantification values of analytes were determined by calculating the ratio of peak areas of transitions of samples normalized to the peak area of the internal standard.

Statistical analysis

Results are shown as the mean (± SD). Statistical significance was calculated by using GraphPad Prism (La Jolla, California). Data was analyzed by student's t-test and $P < 0.05$ was accepted as significant value (***, $p < 0.001$; **, $p < 0.01$; *, $p < 0.05$). At least three biological replicates were done to confirm the results.

Supplementary Figures

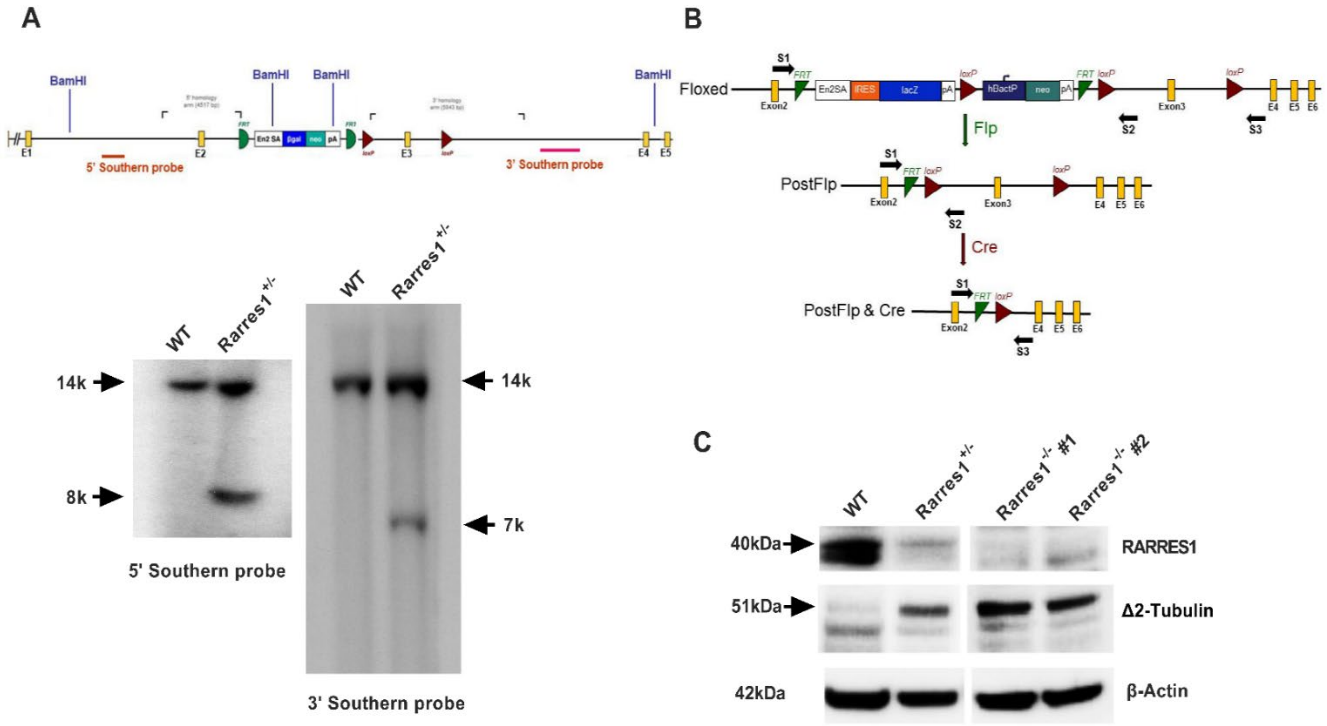

Figure S1. *Rarres1* knockout strategy and validation. (A) Southern blot analysis to show *Rarres1*-gene targeted ES clones, which exhibited 8kb and 7kb band size with *BamHI* digestion followed by hybridization with 5' and 3' flanking probe, respectively. Among 398 clones screened for the homologous recombination, 14 clones were selected with positive signals. Clone 4 and 218 were injected into blastocysts and germline transmission of the floxed *Rarres1* allele was obtained [13, 14]. (B) The *Rarres1* gene targeting strategy for creating *Rarres1*$^{-/-}$ mice in both the 129S1/SvImJ and C57BL/6N backgrounds. Targeting vector design from UCDAVIS KOMP-CSD (ID:39930), PRPGS00072_A_A02, Exon 3 and Diagram of *Rarres1* conditional allele and deletion allele. Primers S1/2 amplify 576bp from the FRT/Flp recombined allele and S1/3 amplify 590bp from the FRT/Flp & Cre recombined allele while wildtype has 464 bp. (C) Western blot analysis of RARRES1 and Δ2-tubulin from the mouse skin of WT and *Rarres1*$^{-/-}$ mice in 129 background strains. RARRES1 functions as an inhibitor of tubulin deglutamylase CCP2 in immortalized cell culture. Thus, levels of deglutamylated tubulin increase upon *Rarres1*$^{-/-}$ mice compared to wildtype (WT) mice.

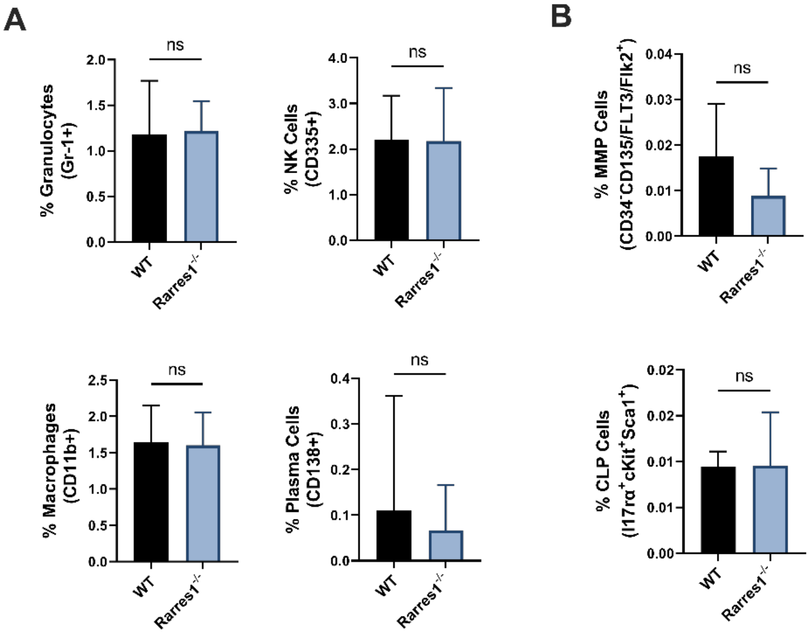

Figure S2. ***Rarres1**$^{-/-}$* **mice have normal numbers of mature splenic blood cells and bone marrow HSPCs.** (A) Flow cytometry percentage of granulocytes (Mac-1/Gr-1+), NK cells (CD335+), macrophages (CD11b/Mac-1+F4/80+), and plasma cells (CD138+) from WT (n = 5) and *Rarres1*$^{-/-}$ (n = 5) spleen. (B) Flow cytometry percentage of MMPs (Lin-Sca1+cKit+CD34-CD135/FLT3/Flk2+) and CLP (Lin-Il7rα+cKit+Sca1+) from WT (n = 5) and *Rarres1*$^{-/-}$ (n = 5) bone marrow. Data shown as mean (±SD). Significance was determined by unpaired two-tailed t-test; *P < 0.05; **P < 0.01; ***P < 0.001; ****P < 0.0001.

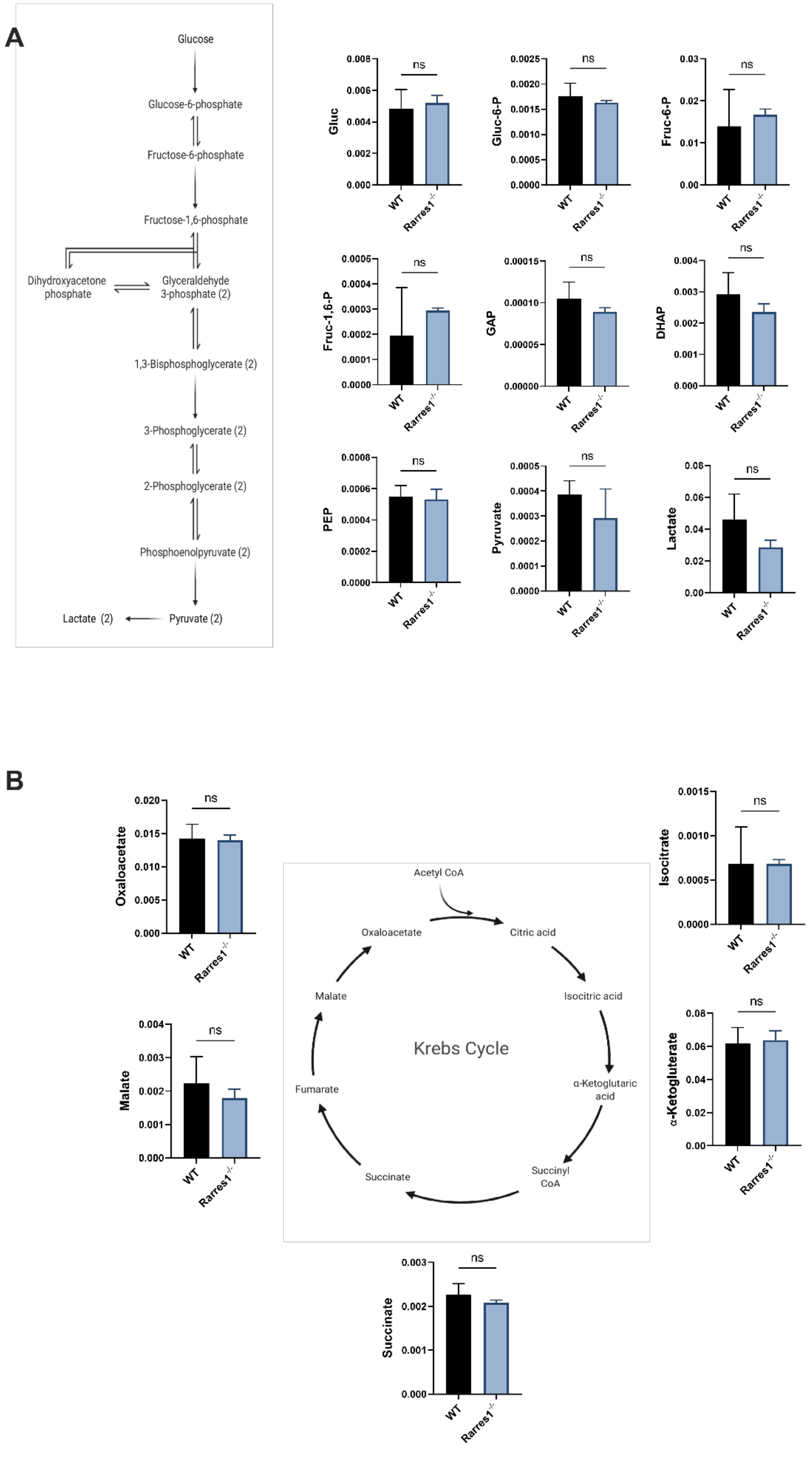

Figure S3. *Rarres1*$^{-/-}$ B cells do not significantly alter central carbon metabolism. (A) Glycolysis metabolites were measured in WT (n = 3) and *Rarres1*$^{-/-}$ (n = 3) 24 hr TD activated B cells. (B) TCA metabolites were measured in WT (n = 3) and *Rarres1*$^{-/-}$ (n = 3) 24 hr TD activated B cells. Data shown as mean (±SD). Significance was determined by unpaired two-tailed t-test; *P < 0.05; **P < 0.01; ***P < 0.001; ****P < 0.0001.

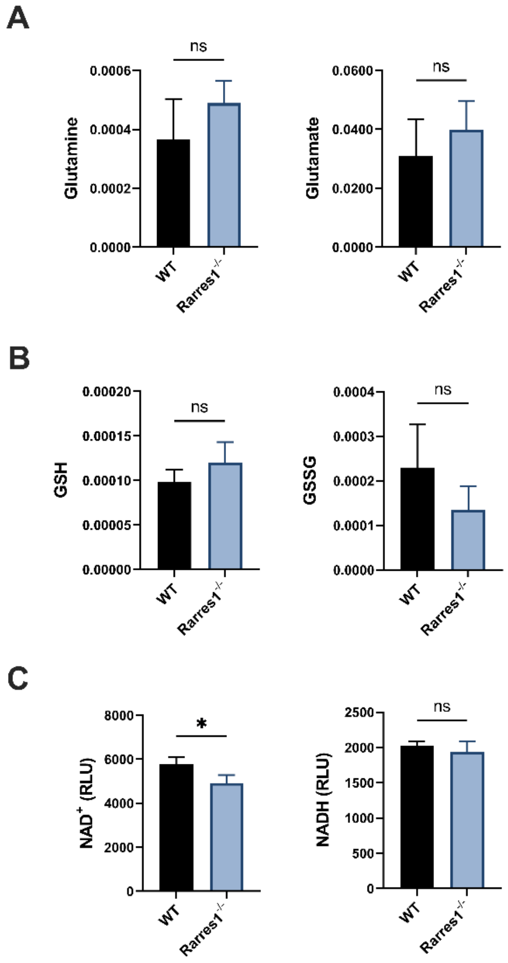

Figure S4. *Rarres1*$^{-/-}$ B cells effects on levels of glutamine and glutamate, glutathione, and NAD+. (A) Glutamine and was measured in WT (n = 3) and *Rarres1*$^{-/-}$ (n = 3) 24 hr TD activated B cells. (B) GSH and GSSG were measured in WT (n = 3) and *Rarres1*$^{-/-}$ (n = 3) 24 hr TD activated B cells. (C) NAD+ and NADH+ were measured in WT (n = 3) and *Rarres1*$^{-/-}$ (n = 3) 24 hr TD activated B cells. Data shown as mean (±SD). Significance was determined by unpaired two-tailed t-test; *P < 0.05; **P < 0.01; ***P < 0.001; ****P < 0.0001.

Supplementary Tables

Table S1. Flow Cytometry antibodies used in the study.

Antibody	Company	Item Number
Brilliant Violet 510 anti-mouse CD19	BioLegend	115545
APC anti-mouse CD3	BioLegend	100235
Brilliant Violet 421™ anti-mouse CD138 (Syndecan-1)	BioLegend	142507
PE/Dazzle™ 594 anti-mouse IgG1 Antibody	BioLegend	406627
Alexa Fluor® 700 anti-mouse I-A/I-E Antibody	BioLegend	107621
FITC anti-mouse IgE Antibody	BioLegend	406905
PE/Dazzle anti-mouse CD335 (NKp46)	BioLegend	137629
Brilliant Violet 711™ anti-mouse/human CD11b	BioLegend	101241
Brilliant Violet 650™ anti-mouse F4/80 Antibody	BioLegend	123149
PE/Cyanine7 anti-mouse Ly-6G/Ly-6C (Gr-1)	BioLegend	108415
Brilliant Violet 421™ anti-mouse Lineage Cocktail	BioLegend	133311
PE anti-mouse Ly-6A/E (Sca-1)	BioLegend	108107
APC anti-mouse CD117 (c-Kit)	BioLegend	105811
PE/Dazzle™ 594 anti-mouse CD34	BioLegend	152209
PE anti-mouse CD135	BioLegend	135305
Brilliant Violet 605™ anti-mouse CD127 (IL-7Rα)	BioLegend	135025
PE anti-mouse/human GL7 Antigen	BioLegend	144607
PerCP/Cyanine5.5 anti-mouse CD95 (Fas) Antibody	BioLegend	152609

Table S2. Primers used in the study.

**Gene**		**Primer Sequence 5' →3'**
sXBP1	F	CTGAGTCCGAATCAGGTGCAG
	R	GTCCATGGGAAGATGTTCTGG
ATF4	F	GGGTTCTGTCTTCCACTCCA
	R	AAGCAGCAGAGTCAGGCTTTC
CHOP	F	CCACCACACCTGAAAGCAGAA
	R	AGGTGAAAGGCAGGGACTCA
BiP	F	TTCAGCCAATTATCAGCAAACTCT
	R	TTTTCTGATGTATCCTCTTCACCAGT
Hsp90aa1	F	AATTGCCCAGTTAATGTCCTTGA
	R	CGTCCGATGAATTGGAGATGAG
Hsp90ab1	F	GTCCGCCGTGTGTTCATCAT
	R	GCACTTCTTGACGATGTTCTTGC
Hspa14	F	TACTCGGAACGTGAACAGGTG
	R	TTCATACCGCAACTTCCCATTT
HSP70-1A	F	TGGTGCAGTCCGACATGAAG
	R	GCTGAGAGTCGTTGAAGTAGGC
HSP70-1B	F	GCGTGGGGGTATTCCAACAT
	R	TGAGACGCTCGGTGTCAGT
RARRES1	F	CTGCGCTGCACTTCTTCAAC
	R	TGCTAAATACCAAGTCCACTTCG
MAPK3	F	TCCGCCATGAGAATGTTATAGGC
	R	GGTGGTGTTGATAAGCAGATTGG
Rn18s	F	GTAACCCGTTGAACCCCATT
	R	CCATCCAATCGGTAGTAGCG

Original whole blot (uncropped blots) for Western/ Southern Blot figures.

Figure 6D

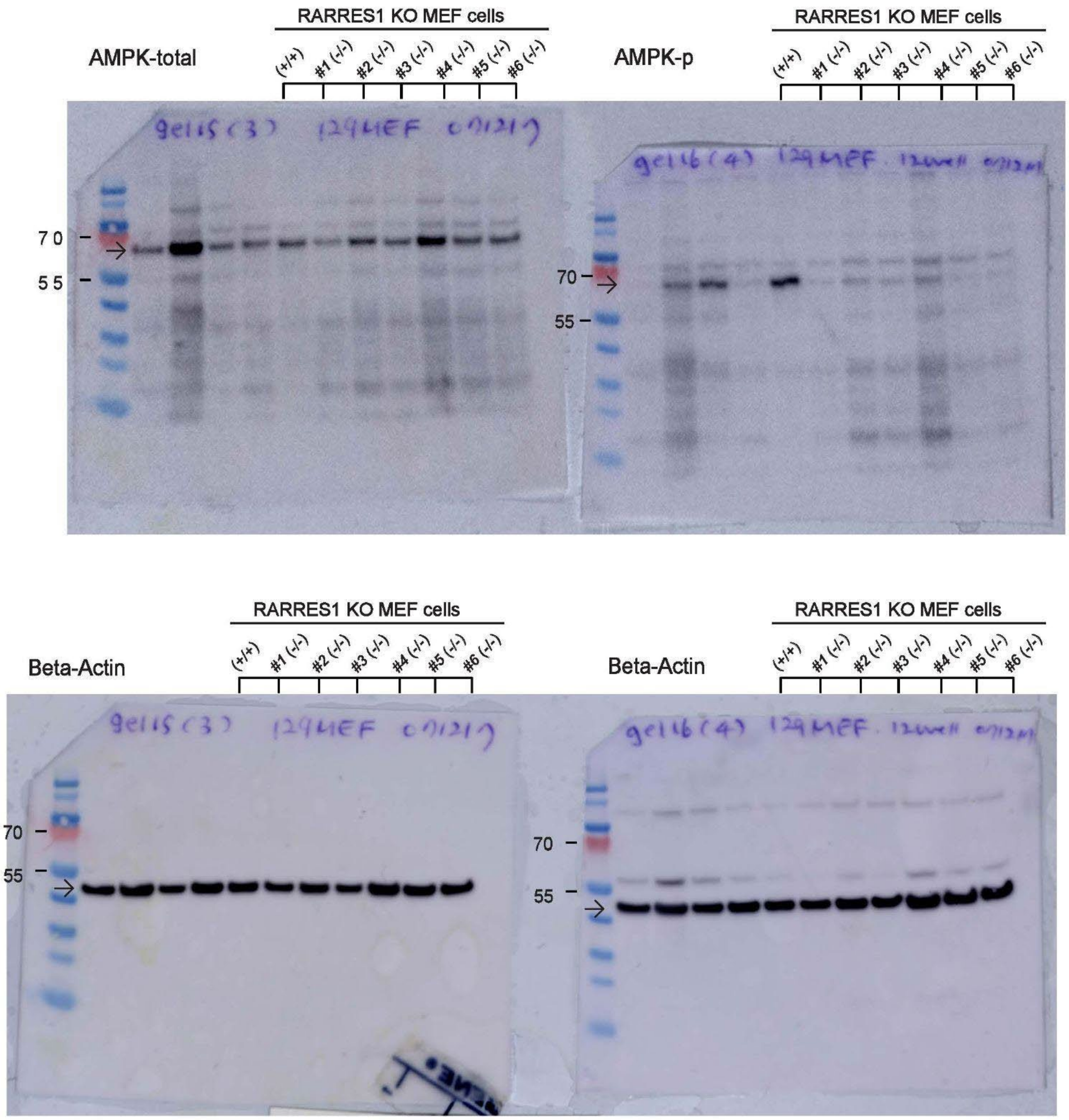

Supplementary Figure 1A

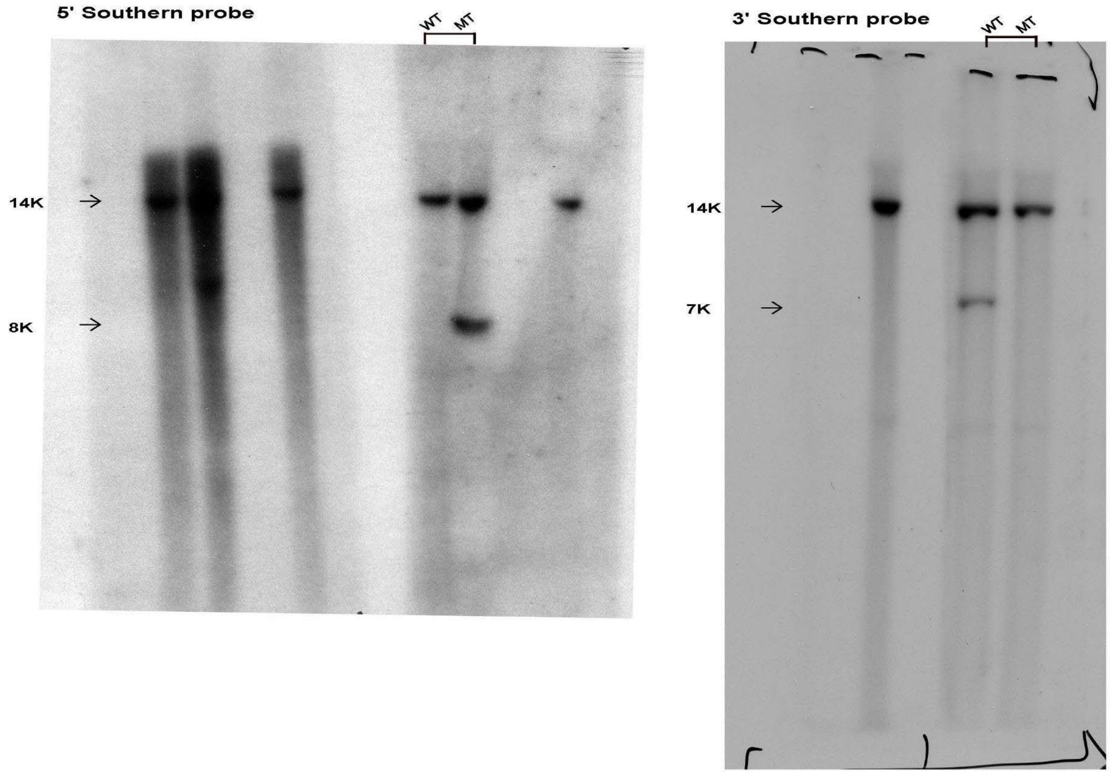

Supplementary Figure 1C

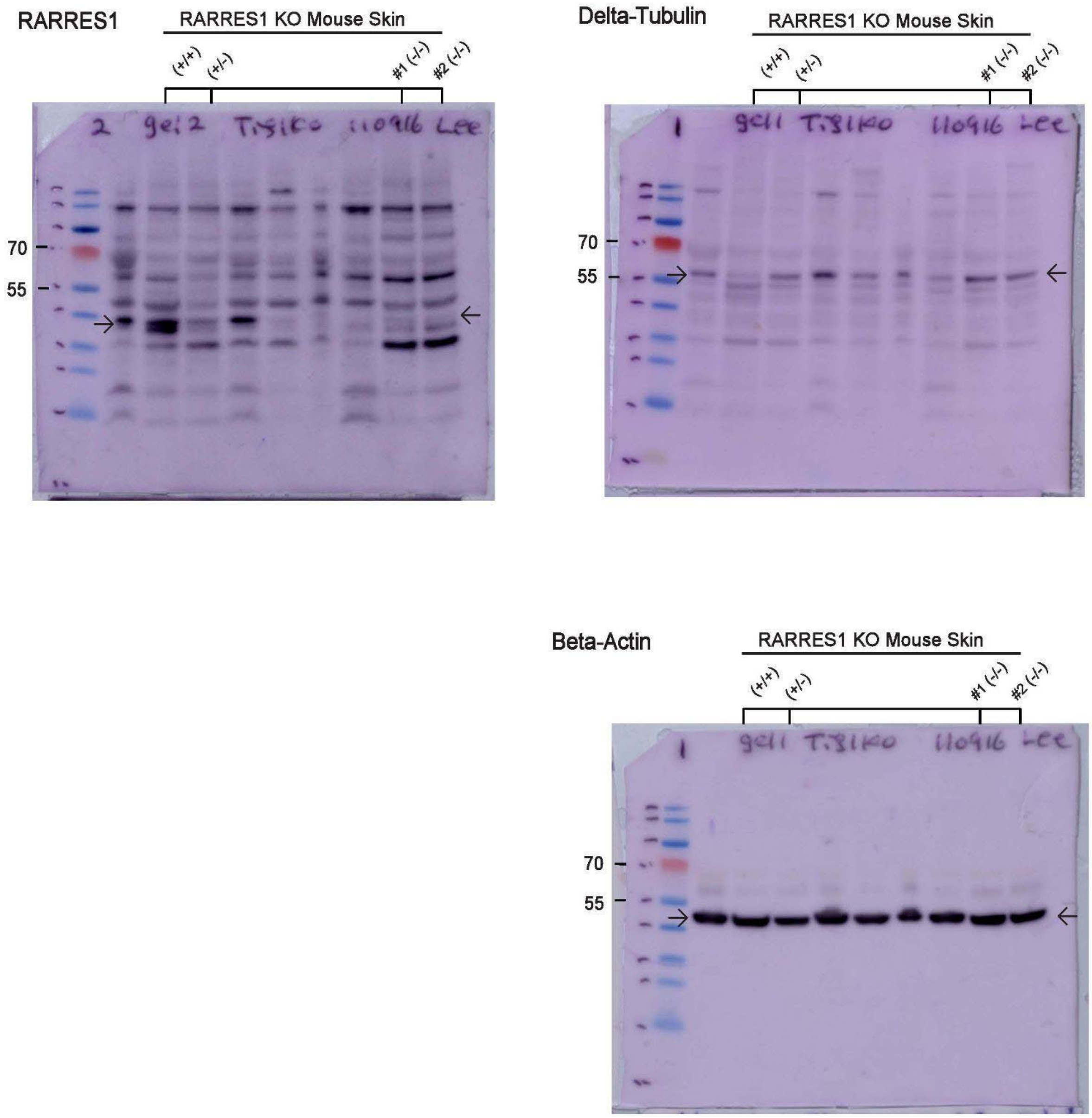

Supplement: Supplementary file 1 — Supplementary methods, figures and tables. [file ijbsv18p2670s1.pdf]
